# An alternative genomic target for proper phylogenetic classification of canine distemper virus (*Morbillivirus canis*)

**DOI:** 10.1007/s42770-026-01876-1

**Published:** 2026-02-22

**Authors:** Sarah Maria van Tol Amaral Guerra, Alice Silveira Becker, José Valter Joaquim Silva Júnior, Otávio Valério de Carvalho, Claudio Canal, Raquel Silva Alves, Rudi Weiblen, Eduardo Furtado Flores

**Affiliations:** 1 Setor de Virologia, Departamento de Medicina Veterinária Preventiva, Universidade Federal de Santa Maria, Av. Roraima, 1000, Prédio 63 A, Rio Grande do Sul 97105-900 Camobi, Santa Maria, Brazil; 2 Programa de Pós-Graduação em Medicina Veterinária, Universidade Federal de Santa Maria, Rio Grande do Sul Santa Maria, Brazil; 3 Sugg Laboratory, Animal Health Research, College of Veterinary Medicine, Auburn University, Alabama, USA; 4 Setor de Virologia, Instituto Keizo Asami, Universidade Federal de Pernambuco, Pernambuco Recife, Brazil; 5 Laboratório NB3 de Neuroimunologia, Universidade Federal de Santa Maria, Rio Grande do Sul Santa Maria, Brazil; 6 Programa de Pós-Graduação em Farmacologia, Universidade Federal de Santa Maria, Rio Grande do Sul Santa Maria, Brazil; 7 TECSA Laboratórios, Minas Gerais Belo Horizonte, Brazil; 8 Bioclin/Quibasa, Minas Gerais, Belo Horizonte, Brazil; 9 Laboratório de Virologia Veterinária, Universidade Federal do Rio Grande do Sul, Rio Grande do Sul Porto Alegre, Brazil; 10 Departamento de Microbiologia e Parasitologia, Universidade Federal de Santa Maria, Av. Roraima, 1000, Prédio 20, Rio Grande do Sul 97105-900 Camobi, Santa Maria, Brazil

**Keywords:** CDV, Phylogeny, Lineage, P/V/C gene

## Abstract

The high variability of canine distemper virus (CDV) highlights the need for adequate genomic analyses to accurately classify viral variants, with whole-genome sequencing (WGS) being the most recommended strategy for this purpose. However, for practical/economic reasons, CDV variants have often been classified by genetic analysis of the hemagglutinin (H) gene, whose suitability for proper lineage identification has been discussed. Herein, we propose an alternative genomic target for genetic classification of CDV in scenarios in which WGS is not feasible. Initially, we designed high coverage primers to amplify an internal region of the gene previously suggested as a potential target for CDV classification (P/V/C gene). The primers were based on 113 WGS of different CDV lineages available (GenBank) and target a 568 bp amplicon. The putative amplicon was then evaluated *in silico* for its ability to reproduce the genetic classification by WGS. After this analysis, we optimized the RT-PCR and evaluated its performance by testing different biological samples previously identified as CDV positive. Amplicons generated from these samples were sequenced and phylogenetically analyzed. Our *in silico* analysis confirmed that the classification based on this amplicon fully reproduces the classification from the CDV WGS. Following this validation, we demonstrated that our primers were able to amplify CDV genetic material from different clinical samples. Finally, the amplicons were easily sequenced by the Sanger method, allowing proper CDV classification. In conclusion, we propose an alternative genomic target for proper phylogenetic classification of CDV, which may be an alternative when WGS is not possible.

**Supplementary Information:**

The online version contains supplementary material available at 10.1007/s42770-026-01876-1.

## Introduction

Canine distemper virus (CDV) (*Morbillivirus canis*), family *Paramyxoviridae* and genus *Morbillivirus* [1], is distributed worldwide and produces a variety of clinical manifestations in dogs, ranging from subclinical to respiratory, dermatological or neurological disease, often with fatal course [2, 3].

The CDV virions are enveloped, contain a helical capsid and a single-stranded, linear, negative-sense RNA genome, 15,690 nucleotides (nt) in length, which encodes six gene products, organized as follows: 3' leader – N – P/V/C – M – F – H – L – 5' trailer [4]. CDV has a high nucleotide substitution rate, which, combined with recombination events, results in multiple viral lineages [2, 5–7]. More than 20 CDV lineages (also termed genotypes) have been described, named based on the geographic location of the first identified sequences. Whenever more than one lineage is identified in a region, they are differentiated by cardinal numbers; and when viruses of the same lineage are identified in more than one region, they are named according to both regions [8].

Classifying CDV lineages is crucial for understanding the distribution of viral strains, monitoring the emergence of new variants and improving diagnostic methods. Additionally, identifying genotypes may provide insights for more effective control and prevention strategies, taking into account the viral genetic diversity [9]. Overall, whole-genome sequencing (WGS) followed by its respective analysis is the most appropriate approach to genetically characterize/identify infectious agents [8]. However, for practical reasons, individual genes or genomic segments have often been analyzed for genetic classification of CDV [10].

Indeed, the identification of CDV variants has been performed by analyzing individual genes or specific genomic regions [11–13]. In particular, the hemagglutinin gene (H) is the most used target for CDV genetic classification, mainly due to its high variability and antigenic role, which would contribute to proper differentiation of CDV variants [14, 15]. In addition, partial sequences of the N and P/V/C genes have also been used for CDV phylogenetic studies [16–19].

Despite the suitability of H analysis for a number of purposes, it was observed that phylogenetic analysis based on this gene may not be equivalent to WGS classification, resulting in some discrepancies. In fact, the P/V/C region proved to be the most appropriate target to reproduce the WGS-based classification [20]. However, performing PCR amplification and sequencing of the entire P/V/C gene for genetic analysis may present challenges not only due to its length, but also because of the difficulty in identifying conserved regions for proper primer annealing.

Considering this, we identified a region within the P/V/C gene whose phylogenetic analysis would reproduce the classification obtained by WGS. We designed high-coverage primers for this target and demonstrated that it may be easily amplified, sequenced and used for CDV classification from different biological samples. Overall, we believe that the strategy described here represents a viable alternative for phylogenetic classification of CDV in scenarios where WGS is not possible.

## Materials and methods

### Study design

Initially, we collected WGS of CDV available in GenBank. These sequences were analyzed to identify conserved regions in the P/V/C gene for primer design, which should also generate an amplicon which would be easily sequenced by the Sanger method. The putative amplicon was phylogenetically analyzed for its ability to reproduce the CDV WGS classification. Subsequently, an RT-PCR assay using these primers was optimized. Finally, the standardized RT-PCR was used for amplification and phylogenetic classification of twenty-two CDV sequences present in clinical samples.

### Data collection

All CDV WGSs available in GenBank (https://www.ncbi.nlm.nih.gov/genbank/) up to April 4, 2024, were collected using the search terms “Canine distemper virus” or “Canine morbillivirus” and filtered to 15,000 and 16,000 nt. After collection, only those identified as “complete genome” were included in our dataset. To avoid possible biases, sequences identified as “recombinant”, “rescue”, as well as those not identified as “isolate” or “strain” or that containing degenerate bases or gaps were excluded.

### Design of primers for amplification-sequencing of genomic targets for CDV classification

The WGS collected were aligned by the Fast Fourier Transformation software (MAFFT, version 7.490) [21], visualized in the Molecular Evolutionary Genetics Analysis software (version 12.0.7) (MEGA 12) [22] and manually analyzed to identify conserved regions in the P/V/C gene for primer design. The sequence of the Snyder Hill vaccine strain (accession number JN896987-1, GenBank) was used as a reference for the location of the P/V/C (nt 1742–3396) gene, considering the coding sequence (CDS) and untranslated region (UTR) regions. The primers were then designed to generate an amplicon of up to 700 bp, which could be easily sequenced by the Sanger method, and to have similar melting temperatures (up to 5 °C difference). Possible nonspecific annealing was analyzed using the Basic Local Alignment Search Tool (BLAST) (National Center for Biotechnology Information, NCBI, https://blast.ncbi.nlm.nih.gov/Blast.cgi).

### Concordance analysis between whole genome versus putative amplicon classification

To assess the suitability of the putative amplicon for CDV classification, two datasets were assembled: one containing CDV WGS and another with the sequence of the putative amplicon without the primer annealing sites. Each dataset was then analyzed phylogenetically according to the best model defined by the MEGA 12 (version 12.0.7) software. Phylogenetic analyses were performed in MEGA 12 (version 12.0.7), using the Maximum Likelihood (ML) method and *bootstrap* with 1000 replicates. Finally, the phylogenetic trees obtained from the two datasets (WGS and putative amplicon) were compared to each other to assess the classification agreement between the two analyses.

### RT-PCR optimization

After phylogenetic analyses and confirmation of the suitability of the amplicon analysis for CDV classification, RT-PCR optimization was performed with the designed primers. The assay was standardized using viral RNA extracted from 100 μL of the supernatant of cell cultures inoculated with CDV 005/15 isolate, using the PureLink™ RNA Mini Kit (Invitrogen). Total RNA was eluted in 30 μL of nuclease-free ultrapure water and the nucleic acid quality was analyzed by spectrophotometry using the NanoDrop™ (Thermo Fisher Scientific). After RNA extraction, complementary DNA (cDNA) was synthesized using the GoScript™ Reverse Transcriptase Kit (Promega), according to the manufacturer's instructions. PCR was performed with Taq DNA Polymerase Recombinant enzyme (5 U/μL) (Invitrogen) for a final volume of 10 μL. The reaction was optimized with different DMSO volumes/concentrations [0.2 μL (2%), 0.4 μL (4%), and 0.8 μL (8%)], different annealing temperatures (50 °C to 60 °C, with 2 °C intervals), and different MgCl_2_ concentrations (1.5 mM, 2 mM, 2.5 mM and 3 mM). The PCR products were analyzed by agarose gel electrophoresis, using GelRed® (Biotium).

### Amplification from and sequencing of the novel target in biological samples

To evaluate the performance of RT-PCR for amplification of CDV in different clinical samples, we tested our assay with twenty-two samples from dogs previously identified as positive for CDV at the Virology Section/UFSM (*Universidade Federal de Santa Maria*, Brazil), TECSA *Laboratórios* (Brazil) or UFRGS (*Universidade Federal do Rio Grande do Sul*, Brazil). Details about the samples, including animal data and collection information, are shown in Table 1.

**Table 1:** Samples analyzed in the study

ID	Biological sample	Collection date	City (Brazil)	Clinic	Vaccination
227/21	Nasal and ocular swabs	08/11/2021	Santa Maria	Respiratory, neurological, gastrointestinal and dermatological signs	No
31/22	Nasal and ocular swabs	12/13/2021	Santa Maria	Respiratory and neurological signs	No
128/21	Nasal and ocular swabs	08/21/2021	Pelotas	Respiratory signs	Yes
130/21	Nasal and ocular swabs	05/11/2021	Pelotas	Respiratory and neurological signs	Incomplete
134/21	Nasal and ocular swabs	06/14/2021	Pelotas	Respiratory signs	NA
122/21	Tissue	NA*	Porto Alegre	NA	NA
200/22	Tissue	NA	Porto Alegre	NA	NA
209/22	Tissue	NA	Porto Alegre	NA	NA
307/24	Rectal swab	10/14/2024	Porto Alegre	No clinical signs	NA
247/24 F	Urine	08/29/2024	Porto Alegre	No clinical signs	NA
247/24 K	Rectal swab	08/28/2024	Porto Alegre	NA	NA
216/24	Urine	08/01/2024	Porto Alegre	NA	Yes
121/24	Rectal swab	06/11/2024	Porto Alegre	Respiratory signs	No
122/24	Rectal swab	06/11/2024	Porto Alegre	Respiratory and neurological signs	No
123/24	Rectal swab	06/11/2024	Porto Alegre	Respiratory and dermatological signs	Incomplete
124/24	Rectal swab	06/11/2024	Porto Alegre	Respiratory signs	Incomplete
125/24	Rectal swab	06/11/2024	Porto Alegre	Gastrointestinal signs	Incomplete
127/24	Rectal swab	06/11/2024	Porto Alegre	Gastrointestinal and neurological signs	Incomplete
120/24	Rectal swab	06/11/2024	Porto Alegre	Dermatological signs	NA
006/676960	Whole blood	01/31/2024	Salvador	NA	NA
021/089389	Urine	01/28/2024	Belford Roxo	NA	NA
031/380473	Whole blood	03/02/2024	Belo Horizonte	NA	NA

*NA data not available

After amplification, PCR products were purified using the PureLink™ Quick Gel Extraction and PCR Purification Combo Kit (Invitrogen) and sequenced in duplicate, both strands, by the Sanger method, using the ACT Gene Molecular Analyses service (Brazil). With the sequencing results, the consensus sequence was obtained by the Staden Package [23] and then phylogenetically analyzed as described in Sect. "Concordance analysis between whole genome versus putative amplicon classification".

## Results

### Primer design

Initially, we collected 257 CDV sequences available in GenBank, of which 144 were discarded because they did not meet the analysis criteria previously described (see Sect. "Data collection"). The dataset with 113 sequences was then used to design the forward (CDV_Snyder Hill_1740_F: 5’- TTAGGACCCAGGTCCAAC - 3’, Tm 62.1ºC) and reverse (CDV_Snyder Hill_2307_R: 5’- TCCCCAGTTAGATGAAGCAT - 3’, Tm 62.4ºC) primers. In detail, primer F annealing sequence covers the last two nucleotides of the N-P/V/C intergenic region up to the first 16 nt of the P/V/C gene (nt 1740–1757 position, Snyder Hill vaccine strain, JN896987-1). Primer R anneals in the P/V/C gene sequence (nt 2288–2307 position, Snyder Hill vaccine strain, JN896987-1).

### Phylogenetic classification based on the novel genomic target is compatible with that of the CDV whole genome sequence

**Fig. 1:**
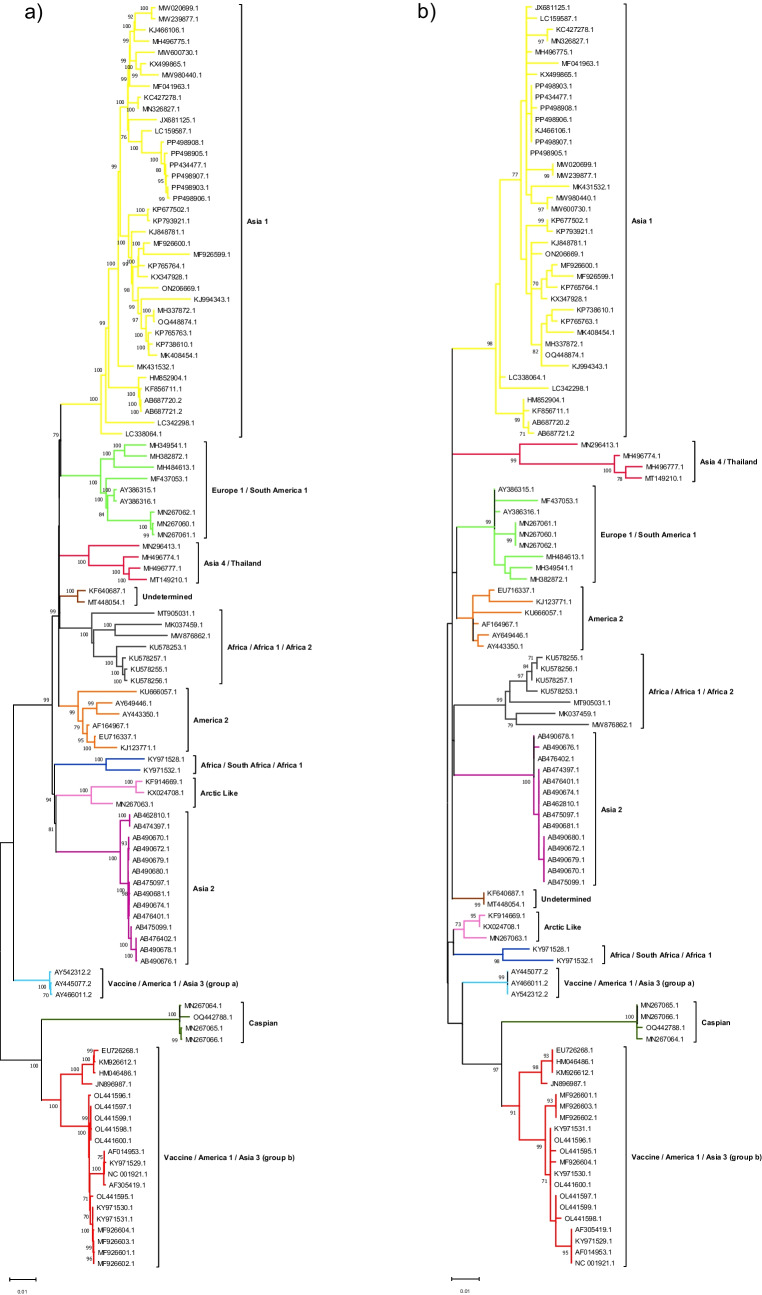
Phylogenetic trees based on nucleotide sequence analysis of the CDV complete genome (**a**) and the novel genomic target/putative amplicon (**b**). Phylogenetic analyses were performed with the Maximum Likelihood method and *bootstrap* of 1000 replicates, using MEGA 12 (version 12.0.7), showing *bootstrap* > 70%. Whole-genome and putative amplicon analyses were performed using the GTR + G + I and K2 + G models, respectively, which were defined by the MEGA 12 (version 12.0.7) software

The CDV WGS from our dataset were classified into ten lineages: Asia 1, Europe 1/South America 1, Asia 4/Thailand, Africa/Africa 1/Africa 2, Africa/South Africa/Africa 1, Arctic Like, Asia 2, America 2, Caspian, Vaccine/America 1/Asia 3 (including group *a* and *b*). Variants that could not be adequately classified into previously identified CDV lineages were classified as “Undetermined”. The identification and naming of CDV lineages were performed following the criteria previously described [20]. Here, all classifications observed in the WGS-based tree were maintained in the phylogenetic analysis of the putative amplicon (Fig. 1).

### RT-PCR assay

**Fig. 2:**
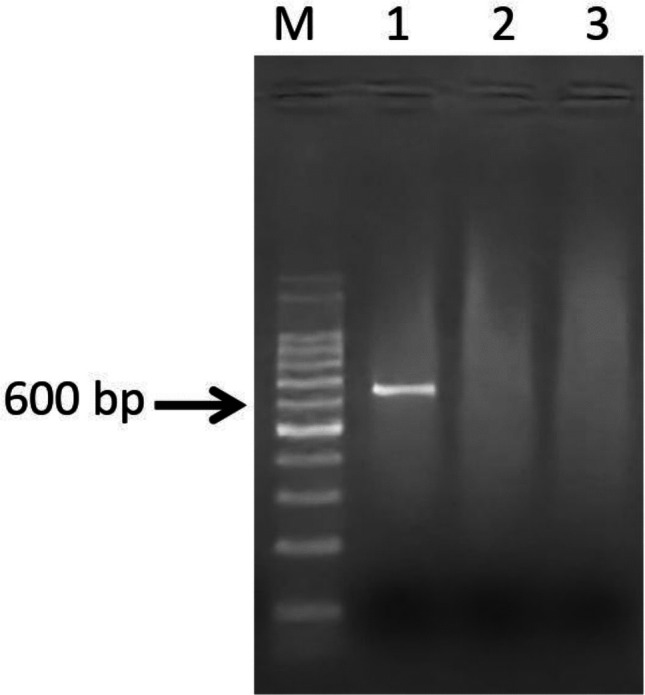
Genomic target amplification for phylogenetic classification of canine distemper virus (CDV). M: Ladder 100 bp (Ludwig); 1: positive sample (568 bp); 2: negative sample; 3: RT-PCR performed with ultrapure water (nuclease-free). The difference in amplicon migration resulting in a band appearing larger than 600 bp is related to the influence of the GelRed dye (Biotium) on DNA electrophoresis

After confirming the suitability of the primer set for CDV phylogenetic classification, RT-PCR was optimized for the following mix conditions: 0.5 µL of MgCl_2_ (50 mM stock), 0.2 µL of dNTPs (10 mM stock), 0.5 µL of each primer (at 10 µM), 0.1 µL of recombinant *Taq* DNA polymerase (Thermo Fisher Science, MA, USA), 1 µL of 10X buffer, 0.8 µL of DMSO, 1 µL of cDNA and 5.4 µL of ultrapure water (final volume 10 µL). The following cycling conditions were used: initial denaturation at 94 °C for 3 min, followed by 40 cycles of 94 °C for 45 s (denaturation), 60 °C for 30 s (annealing) and 72 °C for 50 s (extension), and final extension at 72ºC for 10 min. The PCR products were electrophoresed in a 1% agarose gel and stained with GelRed (Biotium, CA, USA) (Fig. 2).

### Amplification and phylogenetic analysis of CDV from biological samples

**Fig. 3:**
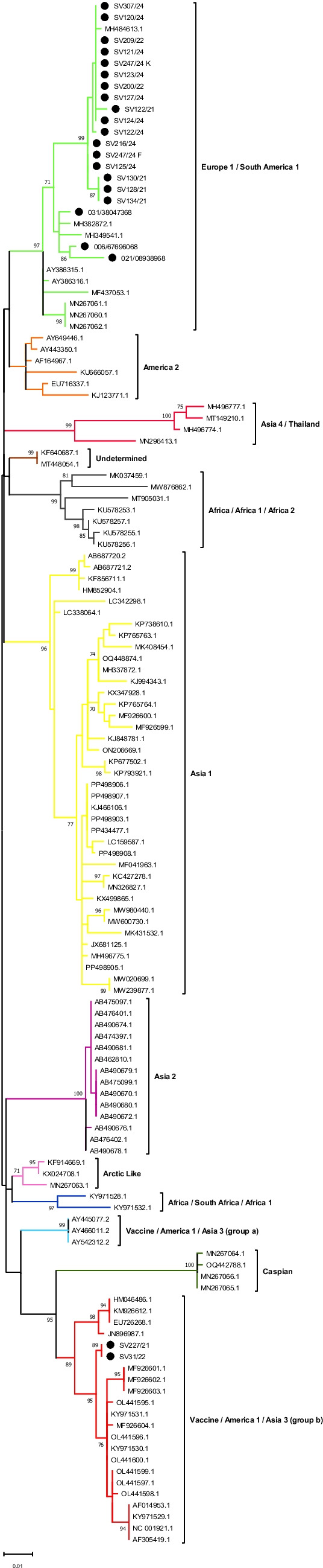
Phylogenetic classification of samples sequenced during the study. Phylogenetic analyses were performed with the Maximum Likelihood method, K2 + G + I model (defined by the MEGA 12 software, version 12.0.7) and *bootstrap* of 1000 replicates, using MEGA 12software (version 12.0.7), showing *bootstrap* > 70%. Samples analyzed in the study are marked with a black circle

Sequences of CDV from 22 clinical samples were amplified in our RT-PCR assay, the amplicons were purified and sequenced with the same primers used for amplification. Among the samples analyzed, twenty clustered as Europe 1/South America 1 and two as Vaccine/America 1/Asia 3 (group *b*) (Fig. 3) (Supplementary file).

## Discussion

An alternative genomic target is proposed for proper genetic classification of CDV lineages/genotypes, circumventing the classification difficulties based on analysis of WGS and the lack of precision encountered by classification based on single genes and/or specific genomic regions. High coverage primers were designed to amplify a region of 568 bp within the P/C/V gene whose amplicon is suitable for genetic analysis and reproduces the analysis of WGS. In addition, the RT-PCR using these primers was able to amplify the target sequences from a variety of clinical samples, allowing nucleotide sequencing by Sanger method and, thus, representing an adequate and viable alternative for many laboratories performing phylogenetic/epidemiological studies of CDV.

The phylogenetic classification of CDV by WGS analysis is inaccessible to many laboratories and research groups; consequently, many CDV genetic classifications have been performed based on the sequencing of individual genes or gene fragments, mainly the H gene [11–15]. Classification based on H, however, may lead to discrepancies compared to WGS analysis. As an alternative, other viral genes have been suggested as potential targets for proper identification of CDV lineages, namely the P/V/C gene [20].

The challenges guiding this study revolve around the amplification of the P/V/C gene and the feasibility of designing high-coverage primers to amplify this region across various viral strains. Amplifying the full-length P/V/C gene can be difficult due to its size and the need to identify conserved regions for adequate primer design. These limitations hamper the amplification and sequencing of multiple samples. To address this issue, an alternative target was proposed: a fragment encompassing the last two nucleotides of the N-P/V/C intergenic region and a specific segment of the P/V/C gene. This fragment/region was selected as a suitable sequence for CDV classification, particularly in scenarios where WGS is not feasible.

Initially, all CDV WGS available in GenBank were collected (*n* = 257) and those that met the inclusion criteria of our study were analyzed (*n* = 113). In the dataset with 113 sequences, which covers the genome of different CDV lineages identified in different countries and regions, primers were designed for P/V/C. This gene was previously suggested as the best target for proper CDV identification, as its analysis reproduces the classification obtained by WGS analysis [20]. As mentioned above, the amplicon generated by the primer set encompasses the last two nucleotides of the N-P/V/C intergenic region and a specific portion of the P/V/C gene. In addition to prioritizing conserved genomic regions, the primers also generate an amplicon with a compatible size (568 bp) for Sanger sequencing.

After primer design, an evaluation was conducted to determine whether the amplicon would be suitable for CDV phylogenetic classification. For this, a comparison was made between the phylogenetic tree yielded by WGS analysis with that of the putative amplicon. Herein, the CDV classification based on the putative amplicon reproduced that of the WGS, confirming the suitability of this target for identification of CDV lineages.

Following the design of primers and confirmation of the suitability of their amplicon for differentiation of CDV lineages, the primers were tested in an RT-PCR assay. The assay was standardized to a final volume of 10 µL, adjusting mix and annealing temperature. The optimized RT-PCR was used for amplification of CDV genetic material from different biological samples (urine, tissues, nasal, ocular and rectal swabs). The assay evaluation of performance with different samples is essential considering the systemic distribution of CDV and the different samples that may be submitted to diagnosis [24, 25].

In addition, the amplicons were easily sequenced by the Sanger method and used for the identification of viral variants. Herein, twenty samples clustered as Europe 1/South America 1 and two as Vaccine/America 1/Asia 3 (group *b*). Importantly, the samples clustered as Vaccine/America 1/Asia 3 (group *b*) are from animals with no vaccination record and share 98.08% and 98.67% nucleotide identity with the Snyder Hill (JN896987.1) and Onderstepoort (NC_001921.1) strains, respectively. In general, these results are in line with those described in previous studies carried out in Brazil. The circulation of CDV belonging to the Europe 1/South America 1 lineage, for example, has been described by the analysis of different genomic targets (H and F genes and WGS), amplified from different biological samples (urine, ocular discharge, blood, brain, serum or rectal swab), collected at different times and different areas in the country [26–32].

In conclusion, we describe an alternative genomic target to proper identification of CDV lineages. The genomic target proposed here may be easily amplified from a variety of clinical samples and may be sequenced by the Sanger method, offering a viable alternative for laboratories where WGS is not possible. Finally, we believe it would be opportune to conduct further studies when more CDV genomes become available, in order to consolidate the suitability of our target for the phylogenetic classification of CDV.

## Supplementary Information

Below is the link to the electronic supplementary material.

Supplementary file1 (FAS 12 KB)
